# Extent, Timing, and Predisposing Factors of Intimate Partner Violence in Sub‐Saharan Africa: A Cross‐Sectional Analysis Using Demography Health Surveys From 2015 to 2021

**DOI:** 10.1002/hsr2.72392

**Published:** 2026-04-19

**Authors:** Abel F. Dadi, Kedir Y. Ahmed, Kayli Wild, Temesgen Yihunie Akalu, Adhanom Gebreegziabher Baraki, Achamyeleh Birhanu Teshale, Tesfa Sewunet Alamneh, Zemenu Tadesse Tessema, Robel Hussen Kabthymer, Koku Sisay Tamirat, Getayeneh Antehunegn Tesema

**Affiliations:** ^1^ Menzies School of Health Research Charles Darwin University Casuarina Australia; ^2^ Addis Continental Institute of Public Health Addis Ababa Ethiopia; ^3^ Rural Health Research Institute Charles Sturt University Orange Australia; ^4^ School of Population Health, Faculty of Health Sciences Curtin University Bentley Australia; ^5^ Geospatial and Tuberculosis Research Team, Telethon Kids Institute Perth Australia; ^6^ Institute of Public Health, College of Medicine and Health Sciences University of Gondar Gondar Ethiopia; ^7^ School of Rehabilitation Therapy Queen's University Ontario Canada; ^8^ School of Public Health and Preventive Medicine Monash University Melbourne Australia; ^9^ Population Health Sciences, Bristol Medical School University of Bristol Bristol UK; ^10^ Department of Medicine, School of Clinical Sciences Monash University Victoria Australia; ^11^ Department of Human Nutrition, School of Public Health Dilla University Dilla Ethiopia; ^12^ School of Rural Health Monash University Victoria Australia

**Keywords:** childbearing age, DHS, IPV, latent class analysis, sub‐Saharan countries

## Abstract

**Background and Aims:**

Reliable evidence on the extent, characteristics, and timing of intimate partner violence (IPV) is crucial for guiding targeted interventions in sub‐Saharan Africa (SSA). This study draws on the most recent demographic and health surveys (DHSs) conducted in SSA to identify approaches for preventing and responding effectively to women experiencing IPV.

**Methods:**

We assessed sociodemographic data, maternal characteristics, and violence from the recent DHS (2015–2021) of 18 SSA countries. We performed a latent class analysis (LCA) to identify women experiencing similar levels of physical, sexual, and emotional violence and to examine their common characteristics. We fit a Cox proportional hazard model to determine predictors of short duration to the first episode of IPV after marriage.

**Results:**

Our analysis included 84,717 women. We identified two distinct classes of IPV experience in SSA: a class with very high (class probability: physical (82.2%), sexual (77.6%), and emotional violence (33.8%) and a class with low violence. Six countries (Sierra Leone, Liberia, Uganda, Mali, Tanzania, and Zambia) constituted more than two‐thirds of women who experienced all forms of violence in SSA. Approximately 25% of women had their first episode of all forms of IPV in their first year of marriage. Two important variables, the husband drinking alcohol and the woman's lack of autonomy, were associated with all forms of IPV and predicted the shortest time to the first episode of IPV after marriage. A husband drinking alcohol alone predicted 61% of IPV cases (area under the receiver operating characteristic curve (ROC) = 0.613; 95% CI: 0.609, 0.616).

**Conclusions:**

This study highlights the importance of comprehensive treatment and prevention efforts that include both criminal justice and public health strategies focusing on women who drink their partner and who have low authority in the first two to 3 years of marriage.

## 1 Background

Violence against women is a major human rights violation and a global public health problem [1], increasingly gaining global attention [2]. Intimate partner violence (IPV) is any abusive behavior by a current or past intimate partner within the context of marriage, cohabitation, or any other formal or informal union and is the most common form of violence against women globally [3]. IPV encompasses physical, emotional or psychological, and sexual violence. Globally, 26% of ever‐married or partnered women over the age of 15 years have experienced physical and/or sexual violence in their lifetime, and 10% have experienced IPV in the past 12 months [4]. In SSA, these rates are even higher, with 33% of women experiencing IPV in their lifetime and 20% experiencing IPV in the past 12 months [5]. Women's experience of IPV starts early in life, increases in middle age, and tends to decline later in life. The coronavirus disease (COVID‐19) pandemic beginning in 2020 increased reports of IPV and brought new attention to addressing violence against women as a public health priority [6, 7].

IPV has significant short‐, medium‐ and long‐term health and physical impacts on women, children, and families and has serious social and economic consequences, with significant geographic and demographic variations [4, 5]. Evidence suggests a link between IPV and adverse maternal, perinatal, child and intergenerational outcomes [8, 9]. Traumatic experiences, including witnessing IPV in early childhood, have been shown to predict greater vulnerability to adverse outcomes in adulthood, including mental illness, difficulties in parenting, and risks of experiencing and perpetrating IPV [8]. Many women who experienced IPV reported consequences such as physical injuries, chronic pain, and suicidal thoughts [10]. IPV is correlated with younger age, greater parity, a low wealth index, lower education levels, drinking or smoking habits, sexual autonomy, and rural residence [11, 12, 13, 14, 15].

Over the last three decades, there has been a strong call to address IPV as a human rights and public health issue in global consensus documents and regional conventions. The 2030 Sustainable Development Goals (SDGs) highlight the importance of eliminating all forms of violence against women and girls in the public and private spheres [16]. Long‐standing advocacy efforts by women's health and rights organizations urge all responsible agencies to engage with IPV and take urgent action. In sub‐Saharan Africa, several efforts have been also considered to reduce IPV and its consequences, including legal and policy reforms, community awareness programs, women's empowerment initiatives, gender‐transformative interventions, and the integration of IPV screening and support into health and social services. Although these approaches have shown some promise, their coverage, accessibility, and effectiveness remain uneven across settings. In addition, many interventions focus on response after violence has occurred, with limited attention to broader structural and contextual drivers of IPV. As such, large, multicounty studies are crucial for improving our understanding of IPV to inform targeted investments in effective and sustainable interventions. Despite the attention given by national and international actors to reducing IPV, limited evidence is available on the extent and characteristics of women with the highest prevalence of physical, sexual, and emotional violence; time to first episode of IPV since marriage; and predictors of IPV in SSA countries. This multicounty study combines DHS data from 18 SSA countries to provide new evidence on the level, timing, and predictors of IPV.

## 2 Methods

### 2.1 Data Source, Design, and Sampling

The DHS is used nationally within low‐ and middle‐income (LMIC) countries to collect cross‐sectional data using a standard questionnaire, ideally every 5 years. This approach is then used to disseminate nationally representative data on a wide range of health and population indicators [17]. The DHS follows a two‐stage stratified cluster sampling design with the first administrative units (e.g., states) as urban and rural strata, a random selection of enumeration areas (EAs) at the first stage, and systematically selected households from EAs at the second stage [18]. We chose SSA countries based on the availability of domestic violence modules, which have been collected since 2015. Our analysis included ever‐married or cohabiting women aged 15–49 years who had complete IPV data. After approval by the DHS data custodians, we accessed the datasets of 18 SSA countries from the DHS program website (https://dhsprogram.com/).

### 2.2 Variables and Measurement

IPV is the outcome variable, and the DHS collects IPV data using standardized questions in the domestic violence module from one eligible woman per household, selected from every second or third household. The DHS uses the modified version of the Conflict Tactic Scale (CTS) to assess the emotional, physical and sexual dimensions of women's exposure to IPV [19]. The potential covariates adjusted in the model included maternal age (categorized as “15–19,” “20–24,“ “25–29,” “30–34,“ “35–39,“ “40–44,” or “45–49“), parity (as a continuous variable), women's level of education (categorized as “no education,“ “primary,“ “secondary,“ or “higher“), marital status (categorized as “married“ or “cohabited,“ occupation status (categorized as “yes“ or “no“), place of residence (categorized as “urban“ or “rural“), wealth quantile (categorized as “very low,” “low,” “middle,” “high,” or “very high”), partner's age (as a continuous variable), partner's level of education (categorized as “no education,” “primary,” “higher” and “unknown,” women's smoking status (categorized as “yes” or “no”), and partner's drinking status (categorized as “yes” or “no”).

We generated two categories of media exposure from a set of three questions: frequency of watching television, reading newspaper/magazine, and listening to radio. The maternal responses to these questions were “not at all, less than once a week, at least once a week, and almost every day.” We categorized a woman who responded not at all to all the questions as having no media exposure (‘no’) or otherwise (“yes”). Women's autonomy was assessed using a set of four questions: i) who usually decides on health care? ii) who usually decides on large household purchases? iii) who usually decides on visits to family or relatives? These questions had four response options: “woman alone,” “woman and husband/partner,” “husband/partner alone,” and “someone else”; and iv) the fourth question, who usually decides what to do with money the husband earns? This question had the options “woman alone,” “woman and partner/husband,” “husband/partner alone,” “husband/partner has no earnings,” and “someone else.” We classified women as “not autonomous” if the responses of the women to the four questions of autonomy were “husband/partner alone” or “someone else,” or “autonomous” otherwise. We controlled the effect of time lag by adjusting for the year in which the DHS was conducted.

### 2.3 Statistical Analysis

We appended sociodemographic characteristics and domestic violence module questions from 18 SSA DHS datasets. We checked for data completeness, calculated a weighted number of study participants, performed a descriptive analysis of variables included in the data, and presented our results using tables and figures. We checked for multicollinearity between explanatory variables using the variance inflation factor (VIF), and a VIF of less than five was used to rule out multicollinearity between predictors of IPV. We separately estimated the prevalence of physical, emotional, and sexual violence for each country.

We then performed a latent class analysis (LCA) to identify the common characteristics of women living in different countries with different levels of physical, emotional, and sexual violence [20, 21]. The LCA is an unsupervised machine learning statistical procedure that is used to qualitatively identify or detect latent (or unobserved) heterogeneity in samples and different subgroups, referred to as latent groups or classes within populations that share certain outward characteristics [22]. The LCA is becoming the most popular social science research method for capturing heterogeneity by integrating person‐centered and variable‐centered analyzes [23, 24, 25]. The LCA assumes that membership in unobserved groups (or classes) can be explained by patterns of scores across survey questions, assessment indicators, or scales. We performed stepwise analysis by fitting a one‐class model, two‐class model, and three‐class model. We used both statistical interpretability and theoretical interpretability to compare the models. A model with two classes fit the data better than the three‐ and one‐class models, as it had low BIC and AIC values as well as better theoretical interpretability [21, 26]. We also extended an LCA to explore the common characteristics of women living in different countries with the highest prevalence of physical, sexual, and emotional violence.

The domestic violence module contained a variable that recorded years to the first episode of IPV after marriage. We used the global Schoenfeld residuals test (*p* value > 0.05) and graphical methods to determine whether the assumption for proportional hazard was met. We then fit the Cox proportional hazard model to identify factors associated with years to the first episode of IPV after marriage. We also estimated the predictive ability of a model with risk factors strongly associated with adverse birth outcomes by calculating the area under the receiver operating characteristic (ROC) curve (AUC) [27]. All analyzes accounted for the complex survey design using Stata's svyset command, and both p‐values and confidence intervals were reported [28]. The DHS dataset for all SSA countries is publicly available with no personal identifiers; thus, ethical approval was not needed.

## 3 Results

Table 1 presents the characteristics of women of childbearing age combined across the 18 SSA countries. Approximately two‐thirds of the sample were from 2016 (28,979 [36.9%]) and 2018 (23,859 [30.4%]) DHSs; were living in rural areas (50,775 [64.7%])); had either no education (19,547 [31.9%]) or completed only primary school (29,331 [37.4%]); reported that they had no media exposure (51,989 [66.2%]); and were working at the time of the survey (52,929 [67.4%]). A very small proportion (798 [1%]) of women included in the survey smoked, while a significant minority (26,753 [35.4%]) of their husbands drank alcohol. The mean (± standard deviation) age of the partner and the number of children the woman had were 38.9 ( ± 11.4 years) and 3.7 ( ± 2.5 children), respectively. Most women were married (60,966 [77.7%]), and the majority (44,929 [57.2%]) lacked decision‐making autonomy.

**Table 1:** Characteristics of woman of childbearing age (15–49 years) in SSA countries included in the study (*N* = 84,717), 2015–2021.

Variables	# of woman with the violence module	weighted # of woman with the violence module	Weighted %
DHS year			
2015	4917	4593	5.8
2016	30,281	28,979	36.9
2017	6401	5551	7.1
2018	25,949	23,859	30.4
2019	3816	3357	4.3
2020	5478	4662	5.9
2021	7875	7500	9.5
Woman's age			
15–19	5394	5019	6.4
20–24	15,375	13,357	17.0
25–29	18,744	16,420	20.9
30–34	16,799	15,008	19.1
35–39	13,107	12,686	16.2
40–44	8884	9221	11.7
45–49	6414	6789	8.8
Husband's age (mean, ±SD)	84,717	78,499	38.9 (11.4)
Residence			
Urban	28,058	27,727	35.3
Rural	56,659	50,775	64.7
Highest education level attained by the woman			
No education	27,028	25,052	31.9
Primary	32,011	29,331	37.4
Secondary	22,119	20,463	26.1
Higher	3559	3655	4.7
Highest education level attained by the husband			
No education	21,290	19,547	24.9
Primary	28,188	25,805	32.9
Secondary	25,625	23,778	30.3
Higher	6490	6376	8.1
Unknown	3061	2955	3.8
Media exposure			
No	54,924	51,989	66.2
Yes	29,793	26,512	33.8
Wealth index			
Very low	18,460	15,286	19.5
Low	17,558	15,801	20.1
Middle	17,165	15,797	20.1
High	16,227	15,814	20.1
Very High	15,307	15,804	20.1
Number of children (mean, SD)	84,717	78,502	3.7 (2.5)
Woman smokes cigarettes			
No	82,782	76,765	99.0
Yes	883	798	1.0
Marital status			
Married	66,328	60,966	77.7
Living with partner	18,389	17,535	22.3
Woman currently working			
No	28,092	25,572	32.6
Yes	56,625	52,929	67.4
Woman's autonomy			
Not autonomous	48,268	44,929	57.2
Autonomous	36,449	33,572	42.8
Husband/Partner drinks alcohol			
No	52,713	48,780	64.6
Yes	29,114	26,753	35.4

Table 2 presents the prevalence of the three forms of IPV in the SSA countries included in the study. Sierra Leone (49.9%: 95% CI: 48.3, 51.6) and Liberia (43.7%: 95% CI: 41.3, 46.2) had the highest prevalence of physical IPV, and Mauritania had the lowest (4.8%: 95% CI: 4.1, 5.7), followed by South Africa (13.4%: 95% CI: 11.9, 15.1). Similarly, Sierra Leone (44.4%: 95% CI: 42.9, 46.1) and Liberia (39.0%: 95% CI: 36.6, 41.4) had the highest prevalence of emotional IPV, and Mauritania had the lowest (10.8%: 95% CI: 9.8, 12.0), followed by South Africa (11.7%: 95% CI: 10.3, 13.3). While sexual violence was generally the lowest type of IPV reported in SSA countries, Burundi had the highest rates (24.2%: 95% CI: 23.1, 25.4), and South Africa had the lowest (3.5%: 95% CI: 2.8, 4.5).

**Table 2:** Prevalence of physical, emotional, and sexual violence by country in SSA (2015–2021).

Countries	Year	Weighted # of woman	Physical violence, 95%CI	Emotional violence, 95%CI	Sexual violence, 95%CI
Burundi	2016–17	5551	37.9 (36.6, 39.2)	22.3 (21.2, 23.4)	24.2 (23.1, 25.4)
Ethiopia	2016	3897	21.9 (20.6, 23.2)	21.8 (20.6, 23.2)	9.5 (8.6, 10.5)
Madagascar	2021	4540	21.4 (20.3, 22.7)	28.2 (26.9, 29.5)	9.4 (8.6, 10.3)
Malawi	2015–16	4171	23.5 (22.2, 24.8)	23.9 (22.6, 25.2)	17.8 (16.6, 19.0)
Rwanda	2019–20	1430	31.9 (29.6,34.4)	28.6 (26.3, 31.0)	12.2 (10.6, 14.0)
Tanzania	2015–16	5873	35.6 (34.4, 36.8)	32.2 (31.0, 33.4)	10.9 (10.1, 11.7)
Uganda	2016	5642	37.2 (36.0, 38.5)	35.3 (34.0, 36.5)	21.2 (20.1, 22.2)
Zambia	2018	5384	34.2 (32.9, 35.4)	25.7 (24.6, 26.9)	13.4 (12.5, 14.3)
Zimbabwe	2015	4593	29.2 (27.9, 30.5)	28.2 (26.9, 29.5)	11.5 (10.6, 12.4)
Angola	2016	7576	31.9 (30.8, 32.9)	25.8(24.8, 26.8)	7.4 (6.9, 8.1)
Cameron	2018	3668	32.5 (31.0, 34.0)	23.5 (22.1, 24.9)	9.1 (8.2, 10.1)
South Africa	2016	1816	13.4 (11.9, 15.1)	11.7 (10.3, 13.3)	3.5 (2.8, 4.5)
Benin	2017–18	3831	18.8 (17.6,20.1)	36.1 (34.6, 37.6)	8.7 (7.8, 9.6)
Gambia	2019–20	1623	28.4 (26.2, 30.6)	22.0 (20.0, 24.1)	5.1 (4.1, 6.3)
Liberia	2019–20	1608	43.7 (41.3, 46.2)	39.0 (36.6, 41.4)	7.7 (6.5, 9.1)
Mali	2018	3130	36.9 (35.2, 38.6)	36.9 (35.2, 38.6)	11.9 (10.8, 13.0)
Nigeria	2018	7847	18.1 (17.5, 18.7)	30.3 (29.6, 31.0)	6.6 (6.2, 7.0)
Sierra Leone	2019	3357	49.9 (48.3, 51.6)	44.4 (42.9, 46.1)	8.0 (7.1, 8.9)
Mauritania	2021	2961	4.8 (4.1, 5.7)	10.8 (9.8, 12.0)	5.9 (5.1, 6.8)

The results of the latent class analysis revealed two classes of women living in SSA countries: a class with a very high prevalence and a class with a comparatively low prevalence of physical, emotional, and sexual violence. The class with a very high prevalence of IPV included women who experienced 82.2% physical violence, 77.6% emotional violence, and 33.8% sexual violence.

**Figure 1:**
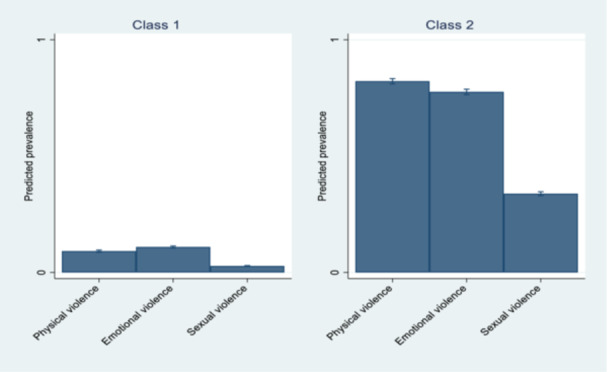
The prevalence of latent classes of IPV experienced by women in different sub‐Saharan African countries (2015–2021).

The class with a low prevalence of IPV included a group of women who experienced 9.1% physical violence, 10.9% emotional violence, and 2.7% sexual violence. The probabilities of women being classified into low‐ and very‐high‐incidence IPV classes were 71.6% (95% CI: 70.5, 72.6) and 28.4% (95% CI: 27.4, 29.4), respectively. (Figure 1 and Table 3).

**Table 3:** Latent class analysis showing the levels of IPV experienced by woman in SSA (2015–2021).

Class name	Types of violence	Probability of experiencing the violence	Probability of being categorized in the class
Low prevalence of IPV group	Physical violence	9.8 (9.1, 10.5)	71.6 (70.5, 72.6)
	Emotional violence	10.3 (9.7, 11.0)	
	Sexual violence	3.0 (2.7, 3.3)	
Very high prevalence of IPV group	Physical violence	82.7 (81.0, 84.2)	28.4 (27.4, 29.4)
	Emotional violence	76.8 (75.2, 78.3)	
	Sexual violence	34.2 (33.0, 35.4)	

Women classified in the very high IPV prevalence class were more likely to have a low education level (AOR: 1.92; 95% CI: 1.52, 2.43), be smokers (AOR: 1.52; 95% CI: 1.25, 1.85), not autonomous (AOR: 1.84; 95% CI: 1.73, 1.96), and drink alcohol from their partner (AOR: 3.62; 95% CI: 3.41, 3.85). (Table 4). Six SSA countries, Sierra Leone, Liberia, Uganda, Mali, Tanzania, and Zambia, had very high incidences of physical, sexual, and emotional violence, ranging from highest to lowest.

**Table 4:** Factors associated with woman in different SSA countries being categorized in the very high IPV prevalence group (2015‐2021).

Risk factors	AOR, 95%CI
Highest education level attained by the women	
No education	1.81 (1.42, 2.32)
Primary	1.92 (1.52, 2.43)
Secondary	1.75 (1.39, 2.19)
Woman smokes cigarettes	
Yes	1.52 (1.25, 1.85)
Woman's autonomy	
Not autonomous	1.84 (1.73, 1.96)
Husband/partner drinks alcohol	
Yes	3.62 (3.41, 3.85)

Approximately 22.5%, 23.6%, and 27.1% of women experienced their first episode of physical, emotional, or sexual violence, respectively, in their first year of marriage. Half of the women experienced their first episode of all types of violence in their first two to 3 years of marriage, while approximately 73% experienced it in the first 5 years. Approximately 4.1%, 3.8%, and 2.8% of women experienced physical, emotional, and sexual violence, respectively, before starting to live with their partners. Several factors have been identified to predict the duration from marriage to the first episode of IPV in each country: maternal age, parity, alcohol consumption, lack of autonomy, smoking status, marital status, education level, wealth index, media exposure, and working status. (Table 5). However, three factors (the husband drinks alcohol, the woman has no autonomy, and parity) consistently predicted a short time to the first episode of IPV in most of the SSA countries included in the study where parity generally had a weak prediction. Husband alcohol consumption alone predicted 61% of the IPV cases (area under the receiver operating characteristic curve = 0.613; 95% CI: 0.609, 0.616). Adding autonomy to the model increased the model predictive ability to 64.1%, and no additional variable significantly changed the model predictive ability. A woman with a husband who drank alcohol had the shortest time to the first episode of IPV. For example, the risk of having a short time to the first episode of IPV among women with a husband who drank alcohol was 2.77 (95% CI: 2.39, 3.21), 2.70 (95% CI: 2.28, 3.20), and 2.32 (95% CI: 1.97, 2.73) times greater among women in Nigeria, Malawi, and Madagascar, respectively. The risk of having a short time to the first episode of IPV among women lacking autonomy was greater in Mali (AHR = 1.90; 95% CI: 1.30, 2.76) and lower in Angola and Burundi (AHR = 1.20; 95% CI: 1.07, 1.36).

**Table 5:** Predictors of time to the first episode of IPV after marriage in different SSA countries (2015–2021).

Countries	Factors associated with time to the first episode of IPV, AHR _(95%, CI)_										
	Maternal age categories			Parity	Husband takes alcohol	Woman has no autonomy	Woman smoking	Unmarried*	Husband education		
	20–24	25–29	45–49						No	Primary	secondary
Burundi	1.29 (1.03, 1.62)			1.04 (1.01, 1.07)	2.23 (1.95, 2.56)	1.20 (1.07, 1.36)					
Ethiopia					1.96 (1.60, 2.41)	1.27 (1.01, 1.61)	2.62 (1.32, 5.19)	2.12 (1.22,3.68)	1.85 (1.01, 3.39)	1.93 (1.10, 3.40)	
Madagascar				1.05 (1.01, 1.10)	2.32 (1.97, 2.73)	1.37 (1.16, 1.62)					1.59 (1.01, 2.50)
Malawi					2.70 (2.28, 3.20)						
Tanzania	1.48 (1.17, 1.88)	1.39 (1.08, 1.79)		1.07 (1.04, 1.10)	2.05 (1.84, 2.29)	1.25 (1.11, 1.41)		0.86 (0.77, 0.97)			
Uganda	1.30 (1.02, 1.66)										
Zambia				1.03 (1.01, 1.05)	1.85 (1.66, 2.05)	1.37 (1.24, 1.51)		0.88 (0.79, 0.98)			
Zimbabwe				1.09 (1.04, 1.14)	1.63(1.44,1.85)	1.49 (1.32,1.68)					
An gola	1.29 (1.03, 1.62)			1.04 (1.01, 1.07)	2.23 (1.95, 2.56)	1.20 (1.07, 1.36)					
Cameron				1.09 (1.05, 1.13)	2.31 (1.90, 2.81)	1.37 (1.17, 1.60)		1.37 (1.17, 1.61)			
Benin				1.12 (1.06, 1.19)	2.07 (1.72, 2.50)	1.62 (1.27, 2.07)	0.26 (0.1, 0.70)	1.69 (1.34, 2.13)			
Gambia				1.08 (1.02, 1.15)							
Liberia			0.48 (0.24, 0.94)		1.81 (1.50, 2.18)	1.25 (1.02, 1.53)					
Mali				1.06 (1.02, 1.10)	2.06 (1.61, 2.64)	1.90 (1.30, 2.76)					
Nigeria	1.43 (1.02, 2.02)	1.52 (1.09, 2.13)			2.77 (2.39, 3.21)						
Sierra Leone					1.70 (1.49,1.93)	1.38 (1.21, 1.58)	1.33 (1.10, 1.61)				
*Note*: * Living together without marriage.											

Countries	Factors associated with time to the first episode of IPV, AHR _(95%, CI)_								
	Wealth index ()				No media exposure	Woman's education			Woman working
	Lowest	Lower	Middle	Higher		No	Primary	Aecondary	
Madagascar		0.71 (0.52, 0.97)			1.27 (1.04, 1.55)				
Malawi	0.67 (0.50, 0.90)								
Rwanda									
Tanzania		1.35 (1.05, 1.75)	1.34 (1.05, 1.70)		1.16 (1.01, 1.33)				
Uganda						1.73 (1.21, (2.47)	2.12 (1.52, 2.96)	1.79 (1.28, 2.52)	
Zambia	1.82 (1.46, 2.27)	1.57 (1.26, 1.94)	1.38 (1.10, 1.72)	1.29 (1.05, 1.59)					
Zimbabwe					1.20 (1.03, 1.41)		1.77 (1.22, 2.57)	1.67 (1.18, 2.36)	1.38 (1.20, 1.58)
Angola									
Cameron									
South Africa									
Benin									
Gambia						2.60 (1.12, 6.02)	2.23 (1.04, 4.80)		
Liberia									
Mali				1.40 (1.01, 1.93)					1.38 (1.18, 1.60)
Nigeria						1.74 (1.03, 2.94)	1.64 (1.05, 2.58)	1.53 (1.07, 2.21)	
Sierra Leone					1.22 (1.10, 1.36)				1.21(1.02, 1.44)
Mauritania									

We also found that three predictors, smoking status, marital status, marital status, and wealth index, differentially predicted the time to the first episode of IPV in different countries. For example, a short duration of smoking predicted a first episode of IPV in Ethiopia (AHR = 2.62; 95% CI: 1.32, 5.19) and Sierra Leone (AHR = 1.33; 95% CI: 1.10, 1.61), whereas a longer duration of smoking predicted a first episode of IPV in Benin (AHR = 0.26; 95% CI: 0.1, 0.70). Similarly, having an unmarried relationship for a short time predicted the first episode of IPV in Ethiopia (AHR = 2.12: 95% CI: 1.22, 3.68), Gabon (AHR = 1.69; 95% CI: 1.34, 2.13), and Cameron (AHR = 1.37; 95% CI: 1.17, 1.61), whereas it predicted a longer time to the first episode of IPV in Zambia (AHR = 0.88; 95% CI: 0.79, 0.98) and Tanzania (AHR = 0.86: 95% CI: 0.77, 0.97).

## 4 Discussion

Preventing violence against women and girls and ensuring responsive and inclusive societies are far‐reaching goals in the SDGs to ensure gender equity [29]. All World Health Organization (WHO) state countries are committed to eliminating all forms of violence against women and girls by 2030 [30]. This study contributes to informing SDG target 17.18, which calls for identifying different causes of IPV through detailed analysis of available data to support national and regional commitments to end all forms of IPV. In this sense, we explored the extent and common characteristics that explain the high IPV prevalence in SSA countries and the high‐risk timing of IPV incidence in married couples to inform targeted investments in eliminating IPV.

Physical violence (up to 49.9%) was the most common type of violence experienced, followed by emotional (up to 44.4%) and sexual violence (24.2%). Sierra Leone had the highest proportion of women with physical and emotional violence, while Burundi had the highest proportion of women with sexual violence. Mauritania is the country with the lowest proportion of women who experienced the three forms of violence, followed by South Africa. We identified a subgroup of countries that had the highest proportion of physical, sexual, and emotional violence and a subgroup of women who experienced all types of violence at the highest rate. Six countries (Sierra Leone, Liberia, Uganda, Mali, Tanzania, and Zambia) constituted more than two‐thirds of women who experienced all forms of violence in the SSA countries. This implies that these subgroups of countries and women could benefit from a common intervention designed based on their shared characteristics. These shared characteristics of women most at risk in these countries included having a partner who drinks alcohol, having a low education level, lacking autonomy, and being smokers. Survival analysis also revealed that these women were more likely to experience all forms of IPV within a shorter period after marriage. All these findings are consistent with other studies conducted in similar contexts at different time points, suggesting that the findings are well‐established evidence requiring immediate and targeted intervention [12, 14, 15, 31].

In this study, approximately one in five married or union women experienced their first episode of IPV in their first year of marriage, while nearly one in two women experienced IPV in their first two to 3 years of marriage. An analysis of IPV in 30 developing countries reported that approximately 38.5% and 67.5% of married women experienced their first episode within one and 3 years of marriage, respectively [32]. Another similar study pooling data from SSA countries reported that half of the women in unions had experienced their first episode of violence within 2 years of marriage [33]. The first few years after marriage, relationships develop, and couples face new ways of living and learn about new environments and experiences, which might bring both challenges and happiness to their lives and might involve different violent activities. This body of evidence reinforces the need for early intervention before couples are married and within the early years of union. Importantly, these interventions should be performed with individuals and specifically target abusive behavior, as couple‐focused therapy can marginalize power dynamics, provide opportunities for abuse to excuse behavior, and increase the risk of further violence [34].

Women living with a partner drinking alcohol were 3.62 times more likely to experience IPV, and their risk of experiencing all forms of IPV could be more than two times greater than that of women whose partner is not drinking alcohol in most of the SSA countries included in this analysis. Furthermore, a husband drinking alcohol alone predicted 61% of IPV cases, and adding autonomy to the model increased the model's predictive ability to 64.1%, making these two variables the most important predictors of IPV. Although the association between partner alcohol consumption and IPV is consistent with the findings of other similar studies [35, 36, 37], recent studies from developing countries have failed to consider partner alcohol consumption as a predictor of a short time to the first episode of IPV [33]. The bidirectional association between IPV and substance use has long been known where substance misuse could be a risk factor or a consequence of IPV victimization and perpetration [38]. This is potentially true because family addiction to substance misuse has significant economic, social, health, and psychological implications both for the family and society, resulting in couples' disagreement and creating barriers in the marital relationship over time.

Our latent class analysis revealed that women with low education and low autonomy were 92% and 84% more likely to experience IPV, respectively, and these women were more likely to experience IPV earlier in marriage in most of the countries. Other studies have also found an association between IPV and women's low education and lack of autonomy [39, 40, 41]. Men who are abusive often feel a deep sense of entitlement, which is further entrenched in patriarchal social systems that give men more power and opportunity than women. Women's autonomy in marriage can be reflected in many situations, such as making decisions on family matters and household expenditures. This study revealed a substantial effect of women's economic autonomy, which has a direct or mediating effect on perpetrating IPV. Intergenerational cycles of violence can be perpetuated through systematic disadvantages and when violence affects the ability to bond with a child and to parent effectively. For example, men are more likely to use violence against their partner when they are insecurely attached or experience attachment anxiety, in which they attempt to regulate this insecurity through dominance and control [42, 43]. Achieving economic autonomy has been a challenge for married women in low‐income countries where the male partner is dominant and is a primary source of income for the family, as explained by attachment theory [44]. While interventions that focus on the broad social goal of gender equality, women's economic independence, and access to justice are vital, there is also important work to be done with men about their early experiences of trauma and how this affects their dominance behavior within relationships [44].

Socioecological theories view IPV as an expression of conflict within the family that can best be understood through the examination of social structures that contribute to the use of violence [45]. On the other hand, feminist theory views IPV as an expression of the gender‐based domination of women by men [46]. The intergenerational transmission model and social learning theory view IPV as a social learning phenomenon in which children grow up in violent families and caregivers themselves as victims of domestic violence and neglect or as perpetrators as they grow to adulthood [47, 48]. The psychological theory of violence looks at IPV as an instinct and a condition of human nature emersed in psychobiological and temperamental vulnerabilities and a result of a damaged psyche that affects self‐regulation, self‐concept, self‐esteem, attachment, and relationships [49]. In general, it is useful to comprehensively view all these theories of IPV from an individual's lifespan perspective, where violence born somewhere in life grows but does not occur among victims or perpetrators but rather passes to the next generation, where its nature, intent, and manifestation vary through the life of an individual. As such, effective treatment and prevention efforts should include both criminal justice and public health strategies starting in early childhood and focusing on multiple generations, but the most effective strategies and timing also need to be developed and tested.

Through this lens, the research presented here provides insights into the individual‐ and country‐level predictors of IPV, illustrating the potential for targeted strategies that are most likely to reach the largest number of people at risk of perpetrating and experiencing violence. These strategies include targeting countries with the highest rates of physical, emotional and sexual abuse; intervening early in relationships; and working directly with men who drink alcohol and women who lack autonomy to address the underlying cycles of trauma that influence behavior over generations.

## 5 Strengths and Limitations

A strength of this study is the use of nationally representative DHS data using standardized data collection procedures, which means that the findings are generalizable within and across SSA countries. We aimed to control changes in IPV incidence over time by including only the most recent surveys and adjusting for the year of the DHS survey. Two factors played the most important role in predicting the probability of IPV, with a predictive power of only 64.1%, indicating that the model did not include other important predictors of IPV, such as maternal mental health, childhood experience of domestic violence and neglect. The DHS used a cross‐sectional study design, which does not permit measurement of causality. The adjustment for complex data analysis uses the primary sampling unit to compute contextual variables that might create bias due to possible misclassification of respondents to incorrect administrative units. Proper class assignment may not be guaranteed because it is based on probabilities, and the exact number or percentage of sample members within each class cannot be determined.

## 6 Conclusion

We identified two distinct classes of IPV experience in sub‐Saharan countries: a class with very high and low physical, sexual, and emotional violence, with probabilities of 28.4% and 71.6%, respectively. Physical violence (up to 49.9%) was the most prevalent type of violence, followed by emotional (up to 44.4%) and sexual violence (up to 24.2%). Six countries (Sierra Leone, Liberia, Uganda, Mali, Tanzania, and Zambia) constituted more than two‐thirds of the women who experienced all forms of violence in SSA countries. Approximately two‐thirds of women experienced their first episode of violence in the first 3 years of marriage. Two important variables, partner drinking alcohol and women's lack of autonomy, were found to increase the risk of IPV and predict the shortest time to first episode of IPV after marriage. Interventions that are most likely to reach the largest number of people at risk of perpetrating or experiencing IPV should focus on individual‐, family‐, and societal‐level factors in high‐prevalence countries to address early trauma experiences, men's drinking, and women's lack of autonomy. These strategies should be used early, prior to the first 3 years of marriage, when the risk of IPV onset is greatest.

## Author Contributions

**Kedir Y. Ahmed:** conceptualization, methodology, validation, data curation, supervision. **Kayli Wild:** investigation, validation, writing – review and editing. **Temesgen Yihunie Akalu:** conceptualization, methodology, validation, writing – review and editing. **Adhanom Gebreegziabher Baraki:** conceptualization, methodology, validation, writing – review and editing. **Achamyeleh Birhanu Teshale:** conceptualization, methodology, validation, writing – review and editing. **Tesfa Sewunet Alamneh:** conceptualization, methodology, validation, writing – review and editing. **Robel Hussen Kabthymer:** conceptualization, methodology, validation, writing – review and editing. **Koku Sisay Tamirat:** conceptualization, methodology, validation, writing – review and editing. **Getayeneh Antehunegn Tesema:** conceptualization, methodology, validation, writing – review and editing, data curation.

## Funding

The authors have nothing to report.

## Ethics Statement

Approval of accessing and the use of DHS dataset was obtained from the Demographic and Health Surveys Program. The analysis used secondary data; there was no contact with the study participants, and informed consent was not available.

## Conflicts of Interest

All other authors declare no competing interests.

## Transparency Statement

The lead author Abel F. Dadi affirms that this manuscript is an honest, accurate, and transparent account of the study being reported; that no important aspects of the study have been omitted; and that any discrepancies from the study as planned (and, if relevant, registered) have been explained.

## Data Availability

All DHS data are available at https://dhsprogram.com/data/available-datasets.cfm. The DHS provides open access to survey data files for legitimate academic research purposes. To initiate the download process, registration is mandatory. Researchers are required to provide their contact information, research title, and a brief description of the proposed analysis. Approval for dataset access is typically confirmed via email. It is important to note that these datasets are third‐party resources and are not owned or collected by the authors, who possess no special access privileges. The analysis files created from these data can be requested from the corresponding author.
